# Risk-proportionate clinical trial monitoring: an example approach from a non-commercial trials unit

**DOI:** 10.1186/1745-6215-15-127

**Published:** 2014-04-16

**Authors:** Catrin Tudur Smith, Paula Williamson, Ashley Jones, Alan Smyth, Simon Langton Hewer, Carrol Gamble

**Affiliations:** 1 Clinical Trials Research Centre, Department of Biostatistics, and North West Hub for Trials Methodology Research, University of Liverpool, 1st floor Duncan Building, Daulby Street, Liverpool L69 3GA, UK; 2 Division of Child Health, Obstetrics & Gynaecology, E Floor East Block, Queens Medical Centre, Derby Road, Nottingham NG7 2UH, UK; 3 Department of Paediatric CF and Respiratory Medicine, Bristol Royal Hospital for Children, Upper Maudlin Street, Bristol BS2 8BJ, UK

**Keywords:** Monitoring, Central monitoring, On-site monitoring, Risk proportionate, Quality assurance

## Abstract

**Background:**

Some level of monitoring is usually required during a clinical trial to protect the rights and safety of trial participants and to safeguard the quality and reliability of trial results. Although there is increasing support for the use of risk-proportionate approaches to achieve these aims, the variety of methods and lack of an empirical evidence base can present challenges for clinical trial practitioners.

**Methods:**

This paper describes the monitoring methods and procedures that are utilised by a non-commercial clinical trials unit which coordinates a range of clinical trials across a variety of clinical areas with different associated risks.

**Results:**

Monitoring activities and approaches should be selected to be proportionate to the risks identified within a trial. A risk-proportionate approach to monitoring is described giving details of methods that may be considered by clinical trial practitioners during the development of a trial monitoring plan. An example risk assessment and corresponding monitoring plan for a low risk (type A in the Medicines and Healthcare Products Regulatory Agency (MHRA) classification system) pediatric trial is provided for illustration.

**Conclusion:**

We present ideas for developing a monitoring plan for a clinical trial of an investigational medicinal product based on our experience. Alternative approaches may be relevant or preferable in other settings based on inherent risk.

## Background

Trial monitoring is defined by the International Conference on Harmonisation of Good Clinical Practice (ICH GCP) as ‘the act of overseeing the progress of a clinical trial and of ensuring that it is conducted, recorded and reported in accordance with the protocol, Standard Operating Procedures (SOPs), Good Clinical Practice (GCP), and the applicable regulatory requirement(s)’ [1]. ICH GCP also states that ‘the purposes of trial monitoring are to verify that: (a) the rights and well-being of human subjects are protected, (b) the reported trial data are accurate, complete, and verifiable from source documents, (c) the conduct of the trial is in compliance with the currently approved protocol/amendment(s), with GCP and with the applicable regulatory requirement(s)’ [1].

The ICH GCP guidance is not specific about which methods should be used but suggests that ‘the extent and nature of monitoring should be based on considerations such as the objective, purpose, design, complexity, blinding, size and endpoints of the trial’ [1]. The guidance highlights a general need for on-site monitoring during different phases of the trial, but recognizes that ‘in exceptional circumstances the sponsor may determine that central monitoring in conjunction with procedures such as investigators’ training and meetings, and extensive written guidance can assure appropriate conduct of the trial in accordance with GCP’ [1]. However, this has been criticized in the literature, with concerns raised that inefficient methods of monitoring are being used unnecessarily in some trials due to misinterpretation of the guidance [2] and a misconception that on-site monitoring is a legal requirement. This has in part led to recent initiatives on risk-adapted approaches to monitoring from the Clinical Trials Transformation Initiative (CTTI) [3], Department of Health [4], Food and Drug Administration (FDA) [5], and the European Medicines Agency (EMA) [6]. These are substantial developments, both for commercial and non-commercial clinical trials, and will provide the potential to reduce costs and increase efficiency. However, there is a lack of empirical evidence to determine which practices best achieve the goals of monitoring stated in ICH GCP [3] and, consequently, heterogeneity in methods of monitoring [7]. Although some empirical evidence is emerging in the literature [8-11], there are very few published examples of monitoring methods that are used in practice, which limits the potential for the sharing of practical experience and expertise to assist clinical trial practitioners developing monitoring procedures to use in practice. The aim of this paper is to describe the risk-proportionate approach to monitoring, with details of central monitoring as well as on-site monitoring methods, currently undertaken at the Clinical Trials Research Centre (CTRC), University of Liverpool, to improve access to, and encourage sharing of practical methods.

## Methods/Design

### Risk-proportionate approach to monitoring adopted at the Clinical Trials Research Centre (CTRC)

The CTRC, based at the University of Liverpool, UK, was established in 2007 and gained full registration status as a United Kingdom Clinical Research Collaboration (UKCRC) clinical trials unit in 2009. The UKCRC has a network of 45 registered Clinical Trials Units (CTUs) which have provided evidence to an international panel of experts of their capability to coordinate multi-centre clinical trials (having overall responsibility for the design, development, recruitment, data management, publicity and analysis of a portfolio of trials), and of robust systems to ensure the conduct and delivery of clinical trials to the highest quality standards. The portfolio of trials that are designed, coordinated and analyzed at the CTRC include pediatric, obstetrics and gynecology, neurology, infection, and dental trials involving investigational medicinal products, devices, and surgical techniques. The trials vary considerably in target sample size, the number of recruiting sites, length of follow up, and experience of research staff at recruiting sites, but all trials would adopt a risk-proportionate approach to monitoring as described in this manuscript.

In terms of the potential risk associated with the Investigational Medicinal Product (IMP), the majority of trials coordinated by the CTRC would be categorized as ‘Type A’ (no higher than the risk of standard medical care) or ‘Type B’ (somewhat higher than the risk of standard medical care) according to the MRC/DH/MHRA Joint Project guidance on Risk-adapted Approaches to the Management of Clinical Trials of Investigational Medicinal Products [4]. Each trial has three oversight committees: the Trial Management Group (TMG) concerned with day to day running of the trial, the Independent Data and Safety Monitoring Committee (IDSMC) to view trial arm comparisons of safety and effectiveness, and the Trial Steering Committee (TSC) that considers the recommendations of the IDSMC and makes the ultimate decision for the continuation of the trial. A separate charter is developed to describe the membership, planned frequency of meetings, roles and responsibilities, and interactions between these committees, which would be referenced within the monitoring plan.

**Figure 1:**
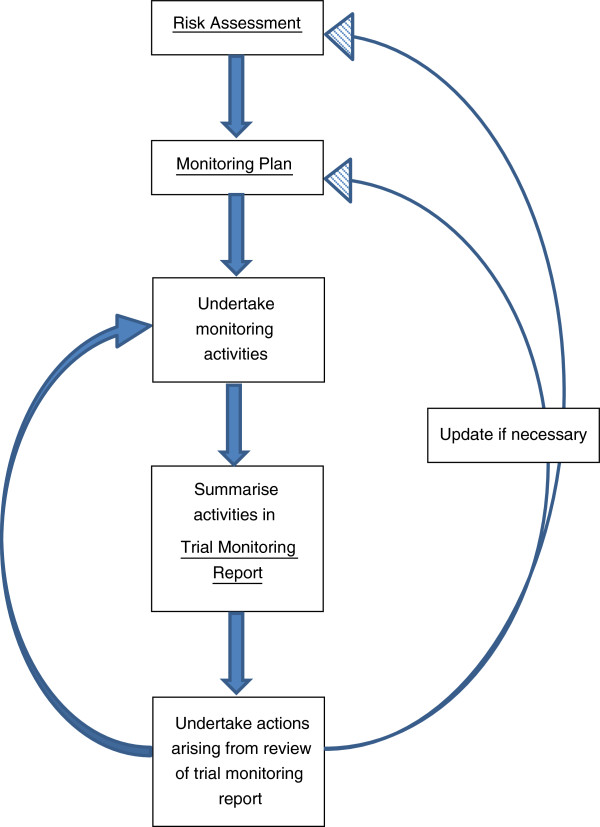
Risk proportionate approach to monitoring at CTRC.

The stages involved in the risk proportionate approach to monitoring adopted by the CTRC are briefly summarized in Figure 1. For each trial, a structured risk assessment is undertaken to identify potential patient, study or organizational hazards (see example risk assessment in Additional file 1).

This requires input from the multi-disciplinary trial team, including statistics, data management, trial management and clinical input. The CTRC Operational Team reviews all CTRC Risk Assessments and final approval of each completed risk assessment is obtained from the CTRC Director, Sponsor and Chief Investigator. The risk assessment form contains both generic and specific hazards and for each hazard it should be documented whether that hazard is, or is not, applicable to the trial. Any additional trial specific hazards should be added to the risk assessment. The total, mean, and overall percentage risk scores are calculated for the trial (using formulae described in Additional file 1) to provide an overall guide and trial risk classification. However, individual risk scores should also be examined closely (using the risk management matrix and key in Additional file 1) to ensure that appropriate strategies are in place for monitoring hazards with particularly high risk scores. The allocation of scores can be subjective and alternative approaches to risk assessment, which do not require the calculation of a numerical score, may be appropriate.

The extent and nature of the monitoring required is determined by the risk mitigating activities to be undertaken and specified within the monitoring plan (example in Additional file 2) with specification of escalation of monitoring activities. The aim of the monitoring plan is to describe how problems that could affect the rights and safety of participants or the reliability of study results can be prevented, or detected in a timely fashion through appropriate monitoring and subsequent corrective action. Regular trial monitoring reports are produced and used to determine when action such as additional site training or on-site visits are required as specified in the monitoring plan. For ‘Type A’ classified trials the monitoring plan would usually describe mostly centralised procedures with triggered on-site visits and would highlight examples of triggers for further action. The risk assessment and monitoring plan may be revised in light of protocol amendments or monitoring reports.

### Developing the monitoring plan

The CTRC monitoring plan is a document which describes all monitoring activities for a particular trial and details planned central monitoring, on-site monitoring, oversight committees, the roles and responsibilities for undertaking activities, and relevant timelines. The plan would also be developed with appropriate consideration for the size of the trial as some monitoring methods may be restricted for small trials. Examples of monitoring activities that would be included within the monitoring plan are described in the following sections. An illustrative example is provided in Additional file 2 which relates to a pediatric CTRC ‘Type A’ Trial of Optimal TheRapy for Pseudomonas EraDicatiOn in Cystic Fibrosis (TORPEDO-CF). The summary protocol and corresponding risk assessment are also provided (Additional files 1 and 3).

### Responsibilities

The Sponsor has ‘ultimate responsibility for the quality and integrity of the trial data’ [1] but may delegate activities whilst maintaining appropriate oversight. The multidisciplinary nature of the TMG and the multilevel nature of monitoring approaches demand the need for individual responsibilities and expectations to be clearly defined within the monitoring plan. The CTRC monitoring plan development team includes the Trial Coordinator (TC), Trial Statistician (TS), Trial Monitor (TM) (if relevant) and Chief Investigator (CI) of the trial. This development team structure is regarded as the minimum requirement with input sought from additional expert members, such as a pharmacist, where specific hazards have been identified. The TC takes overall responsibility for coordinating the production of the monitoring report. The CTRC Senior Management Team (SMT), which consists of the CTRC Director, Head of Statistics, Head of Trial management, Head of Information Systems, Quality Assurance Manager, and Head of Data Management, review and sign off the monitoring plan for each trial before implementation.

## Results and discussion

### Trial monitoring approaches

There are essentially two approaches for monitoring clinical trials: central monitoring, and on-site monitoring, with the monitoring plan providing a summary of the practical procedures and reporting summaries required to undertake these activities.

### Central monitoring

Central monitoring involves centralized procedures for the quality control of trial data. Responsibility for each section of the monitoring report should be specified within the monitoring plan. There are numerous central monitoring procedures that may be considered for an individual trial. Some approaches involve using statistical methods (central statistical monitoring) to explore patterns in the accumulating data, some involve monitoring results of automated validation checks that may be built into the data management facility, some may involve the central review of forms submitted to the CTRC, and some may involve a comparison of performance across participating sites. Defining an ‘acceptability threshold’ , such that the crossing of this threshold would trigger action, may also be helpful to guide decision making for some monitoring activities (for example see Section ‘missing primary outcome data’) These central monitoring methods are used to assure the quality and reliability of trial data, protect the safety of trial participants, detect trial conduct problems at particular sites, and can be useful for detecting fraud [12]. Listed below and in Additional file 2 are examples of central monitoring procedures that would usually be considered for CTRC trials, although it is recognized that this is not an exhaustive list.

### Review of consent forms

Documenting that the consent process has taken place and that trial participants have been fully informed about the trial is an essential process to safeguard the rights of trial participants. In general, copies of completed consent forms are faxed to the CTRC within seven days of completion by participating sites. Each form is then checked using a consent form checklist to ensure that each form has been fully and accurately completed and returned from sites within a reasonable time frame. Any issues identified through this routine review of consent forms is summarized in the trial monitoring report to enable the overall site specific issues to be reviewed. As patient signatures are included on completed consent forms, all faxed copies are kept securely in a locked cabinet, separate from other trial documents. Patient names are not routinely recorded or linked in any way with further data which may be collected during the trial. Furthermore, the patient consent form explicitly requests permission for the completed consent form to be passed to the CTRC for the administration of the study.

### Checks across individual sites for problems

In multi-centre trials, data from one site can be compared with data for the whole trial to compare performance and identify patterns which may indicate that action is required. The key data items to be compared across sites should be identified after consideration of the risk assessment and listed in the monitoring plan. Methods for statistical monitoring such as exploring digit preference, rounding, or unusual features of a frequency distribution (such as outliers, inliers, or atypical degrees of skewness or kurtosis) which have been described [2] for the detection of data fabrication or data falsification, may be useful when considering the comparison of data across sites. Graphical approaches such as site-specific box and whisker plots of key continuous variables, or graphical approaches described in later sections, can help with interpretation of site comparisons and facilitate decision making. However, it should be noted that differences in participant characteristics across sites may explain apparent differences for certain types of variables. Furthermore, the application of some methods for statistical monitoring are limited in small trials and the issue of multiple testing needs to be considered carefully if significance tests are to be performed.

### Recruitment and retention

Recruitment of participants below the expected or predicted recruitment rates can jeopardise the power of the study, impose time delays and financial penalties on the trial, and can lead to premature trial closure. Frequent monitoring of recruitment data will help to identify whether the target sample size can be achieved within the expected timeframe, and any appropriate corrective action that may be required.

The frequency of non-eligible patients and eligible patients who do not provide consent (with a summary of the reasons) compared across sites can give valuable information about the recruitment process, including restrictive eligibility criteria or issues in its application or interpretation. Comparison of site specific retention rates (percentage of patients at a site that have withdrawn) can highlight sites where there may be issues with following the correct consent process.

**Figure 2:**
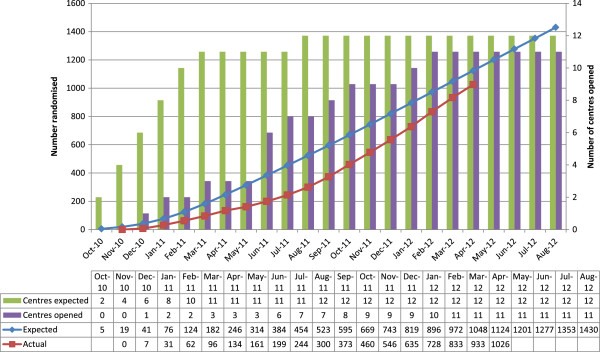
Overall recruitment graph.

The benefits of monitoring recruitment by site include identification of sites which are recruiting well to determine successful recruitment initiatives relevant to other sites, additional site-specific support can be provided for poor recruiting sites or poor recruiting sites can be closed and resources redistributed appropriately. Early identification is essential to allow adequate time for planning additional trial-specific and site specific resources. An example graph showing overall recruitment and site openings is presented in Figure 2. Similar site specific graphs or graphs showing site specific recruitment rates per unit of time can also provide valuable information.

### Randomization system

Adequately concealed randomization systems eliminate selection bias and produce groups of participants that are balanced with respect to known and unknown prognostic factors. All randomized trials should be monitored to ensure that the randomization system is working correctly and that participants are being randomized properly. These checks would be undertaken by the TS and would usually coincide with the preparation of data monitoring reports presented to the IDSMC. However, a minimum check should be scheduled during early stages of recruitment to promptly identify problems and apply corrective action. The risk assessment may indicate that more frequent monitoring is required if, for example, randomization envelopes or a web-based minimisation procedure are being utilised. The appropriate checks to undertake will depend on the method of randomization but would include a summary of baseline characteristics and number randomized between treatment groups (usually presented in the IDSMC report), as an imbalance could indicate a problem with the randomization system. If stratification has been used in the randomization process the number randomized in each treatment group across stratification variables should be summarized. Depending on the implications for unblinding, details of such randomization checks may not be included in the trial monitoring report beyond stating that the check was conducted with results held confidentially by the trial statistics team. In addition, the randomization numbers should be checked to ensure that they have been allocated in chronological order with any missing randomization number accounted for with appropriate explanation. The correct implementation of the randomization process at sites is crucial. Any site specific randomization problems should therefore be summarized in the trial monitoring report with only a minimum number of errors required to trigger further action.

### Missing primary outcome data

**Figure 3:**
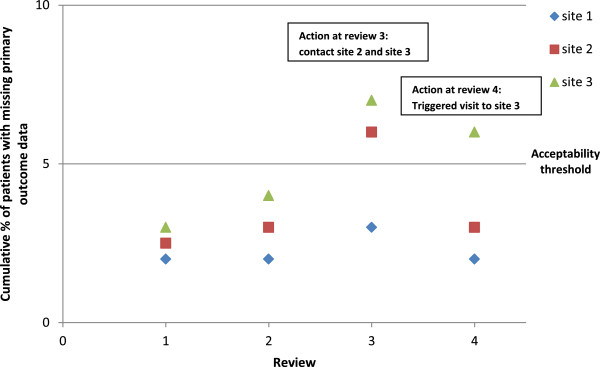
Example of acceptability threshold graph for missing primary outcome data.

Missing primary outcome data can affect the reliability of trial results, particularly when there is a substantial amount of missing data or if reasons for missing data are related to treatment or outcome. Procedures to minimise the occurrence of missing data should always be considered and incorporated into the trial design, and repeatedly highlighted to staff throughout the trial. To allow for the potential impact on power it is usual practice to inflate the estimated sample size to allow for a certain percentage of missing data. This inflation factor may be a reasonable upper value to define an acceptability threshold for missing primary outcome data to guide decision making and appropriate further action during monitoring activities. The cumulative percentage of participants with missing primary outcome at each site can be plotted (Figure 3) at various time-points, which need not necessarily be equally spaced. This graphical approach can facilitate interpretation and help easily identify sites which cross the acceptability threshold (5% is used in Figure 3), or which repeatedly remain at an unacceptably high level. The reason for missing primary outcome data should be identified and summarized along with the graph. Any missing primary outcome data should be routinely queried with the site as soon as possible. If the cumulative percentage of participants with missing primary outcome data crosses the acceptability threshold the site should be contacted to discuss and reiterate the importance of complete data for this outcome. A subsequent site visit should be arranged if the problem is still apparent in the next trial monitoring report.

### Patient safety

**Figure 4:**
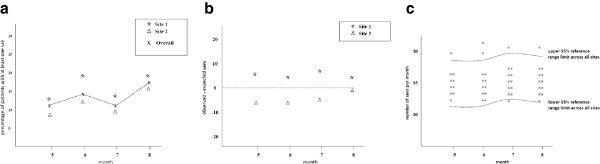
**Example graphical approaches(hypothetical data)for comparing serious adverse events (SAEs).(a)** percentage participants with at least one SAE, **(b)** discrepancy in SAE event, **(c)** SAE rate at site 1.

The pharmacovigilance plan is specified in the protocol and considered appropriate to the risks identified in the trial and what is known about the interventions and conditions under study [13,14]. The IDSMC is responsible for monitoring patient safety throughout the trial and this would usually be done through a regular review of accumulating data. The trial monitoring plan would usually include details of safety reporting indicators (identified from the risk assessment) that are compared across sites to identify whether events are being identified and reported consistently, and in a timely manner. Example graphical approaches for comparing safety reporting indicators are provided in Figure 4 (hypothetical data used for illustrative purposes only) where the key data item of interest is serious adverse events (SAEs).

In Figure 4a, the cumulative percentage of participants with at least one event across the trial as a whole, and at each site, are plotted together against some measure of time (month in this example) which need not be equally spaced. This can be used to check for under or over reporting of SAEs at sites, and is a useful approach if a small number of events of this particular type (SAEs in this example) are expected, and most likely to occur infrequently per participant. Large discrepancies can be discussed and monitored closely over time to identify whether any patterns persist which may trigger further action.

The approach in Figure 4a does not account for the length of follow-up of each individual participant. If there is variability in participants’ length of follow-up and follow-up time is expected to increase the likelihood of an event such as an SAE, the approach summarized in Figure 4b may be preferable. Here, the overall event rate for the trial (sum of all events divided by the sum of all follow-up for the trial) is calculated, along with the expected number of events per site (sum of all follow up at a site x overall event rate for the trial). The difference between the actual and expected number of events at each site is plotted against time (Figure 4b).

If the event of interest is expected to occur frequently with multiple events per participant, such as SAEs in a cancer trial, the plot in Figure 4c may be most appropriate. Here the overall mean number of SAEs per participant per unit time (such as month) is calculated across all sites (sum of all SAEs divided by the sum of all follow-up) with a reference range (for example a 95% reference range = mean ± 1.96 x sd, where sd = √mean). It may be necessary to use a narrower reference range for early monitoring, or perhaps plot a 95% and 90% reference range on the same graph. The number of SAEs per unit time for each individual participant (sum of all SAEs for participant j divided by the sum of all follow-up for participant j) is plotted along with the overall mean number and reference range. This may be presented on a single plot highlighting outliers (with different symbol for each site) above or below the reference range or as site specific graphs comparing the distribution of events. The site specific percentage of participants with SAE rate outside the reference range can also be calculated (or plotted over time) to guide interpretation. The trial monitoring plan should specify guidelines to follow to determine appropriate action if the SAE rate crosses a particular threshold (such as upper and lower limits of the reference range).

### Protocol deviations

Key protocol deviations that might occur during the trial should be identified by the TMG (in conjunction with the risk assessment) and summarized in the trial monitoring plan. The TORPEDO-CF monitoring plan (Additional file 2) provides an example of a protocol deviation table, listing examples of potential deviations of important protocol specifications such as eligibility criteria, treatment regimens and study assessments. For each item, the potential protocol deviations that may occur are listed and graded in terms of the impact such a deviation would have on patient safety and the reliability of trial results. This is generally graded as major (potentially major impact either through introduction of bias to study results or effect on safety) or minor (potentially minor impact on patient and not likely to introduce bias), but the grade may vary according to the degree of deviation observed. Justification is also provided for the assessment of the impact of each potential protocol deviation. All protocol deviations that occur during the trial should be summarized within the trial monitoring reports with particular focus on major deviations. If a particular deviation occurs frequently this may indicate the need for a protocol amendment. The graphical approaches described in Figure 3 and Figure 4 may be appropriate for the comparison of protocol deviation rate to identify sites which have more frequent deviations from protocol thereby requiring further training or on-site visits.

### Case report form (CRF) completion monitoring

Paper CRFs are routinely checked (manually or electronically) when received to ensure that they are signed by authorised personnel. An electronic CRF tracking form keeps a record of CRFs received into the unit. Full details of the CRF tracking system should be included in the data management plan. A summary of data from the CRF tracking system, in particular missing CRFs, delay submitting CRFs (paper or electronic) to CTRC, completeness of reporting, and delay responding to queries can be generated by site or other alternative as appropriate. If an individual site consistently fails to keep up to date with CRF (paper or electronic) completion and timely submission to CTRC, the TC should work with the site to develop a plan to correct this issue.

### Validation of data

Range and consistency checks can be used to identify unlikely or implausible data and would usually be programmed into the trial database of all CTRC trials during database development. All validation and consistency checks are recorded in the trial data management plan and referenced within the trial monitoring plan. External sources of information may also be available to check the validity of certain data variables. For example, date and cause of death can be verified by using death registry data such as that recorded by the Office of National Statistics in the UK.

Visit dates, or other important dates can be routinely examined centrally to check for consistency and accuracy. For example, in some trials a weekend clinic visit would not be expected to occur and may indicate a data entry error or in some rare cases could suggest fraud. Visit date checks can also be used to identify visits which have occurred outside of protocol timelines or identify visits that have been missed. In larger trials it may be preferable to present site specific scatter plots of actual date of visit compared to expected date of visit and compare the percentage of visits outside the permitted visit window across sites. Finally, it is possible to identify inconsistent dates by cross checking different information within, and across CRFs for a patient.

Summarising the results of range, consistency and validation checks across sites can provide useful information about compliance with the trial protocol and procedures, indicate resource issues at sites, and can be helpful supporting information when deciding whether site visits may be required.

### Pharmacy compliance with IMP handling procedures (clinical trials of IMPs only)

Checks should be performed of the suitability of the environmental conditions (such as a central review of temperature logs), suitability of the product (expiry dates, potential degradation of the presentation) and checks that all medication can be accounted for via dispensing logs, checking that any medication returned by a participant matches the amount dispensed minus the amount consumed.

### Quality assurance of statistical analyses

All interim and final statistical analyses are undertaken in accordance with the relevant standard operating procedures and statistical analysis plan. The derivation of the primary outcome, corresponding statistical analyses, and safety data are independently programmed by a CTRC statistician independent to the trial. These independent analyses are cross checked with those undertaken by the trial statistician. This is particularly important in trials which include a primary outcome with a complex definition that depends on multiple variables, requiring substantial programming.

### CTRC internal performance monitoring

In addition to monitoring the performance of sites involved in the trial, it can often be important to monitor performance of the coordinating trials unit. One example would be to review the data entry process by comparing data entered by trials unit staff on the database against the data recorded on the paper CRF. This would usually be undertaken for all randomization related data and primary outcome data as a minimum with additional key data items identified as important from the risk assessment. These reviews are useful to (a) assure data accuracy, and (b) to identify whether data entry staff at the CTRC require further training or support. It may also be relevant to review other measures such as the time between receipt of paper CRF and completion of data entry, or number and type of queries raised, to allow potential delays in the system to be highlighted so that appropriate action can be taken as early as possible, minimising the negative impact on accumulating trial data. A summary of these internal performance reviews should be included in the trial monitoring report at the relevant time point, together with any required action plan.

### Other issues

Other important hazards that require central monitoring should be identified from the risk assessment and appropriate procedures listed in the monitoring plan. For example, trials which rely on a blood test to guide treatment dosing would benefit from monitoring the number of invalid or missing blood samples across sites; trials using variable dosing of treatments may summarize dose (for example median, interquartile range, minimum and maximum) across sites which could identify inappropriate dosing that may have an impact on patient safety; in blinded trials the emergency unblinding rate should be compared across sites with appropriate measures in place to assure that the correct procedures have been followed.

### On*-*site monitoring

On-site monitoring involves a member of the trial management team visiting an individual site to undertake monitoring activities. On-site monitoring may either be ‘routine’ in accordance with the monitoring plan, or may be ‘triggered’ as a result of the TMG review of trial monitoring reports. The rationale, timing, frequency and activities to be undertaken during routine on-site monitoring visits should be decided by the TMG in conjunction with the risk assessment, with appropriate consideration given to the rate of participant recruitment at each site, the complexity of trial procedures, and experience of the site in conducting clinical trials.

If routine on-site monitoring is planned, the first site visit should ideally occur during the early phase of the trial following recruitment of the first few participants at the site to allow the monitor to pick up early issues, confirm accurate interpretation of the protocol and data management procedures, and address any unresolved queries or problems in a timely manner. Site visits will normally involve the Principal Investigator (PI) and site research nurse/practitioner but may involve any or all of the individuals involved with the trial at the particular site. This may include for example a research radiographer, trial pharmacist, or data manager at site.

During routine site visits, focus would tend to be on making sure that trial procedures have been conducted in accordance with the trial protocol and according to the principles of GCP under the UK regulations. Any issues identified are discussed with site staff and explanations or remedial action documented within the site monitoring visit report which is sent to the site and also included in the TMG trial monitoring report. Routine on-site visits provide an opportunity for trial management staff to offer mentorship and additional training for site staff to ensure that procedures are being followed as outlined within the protocol.

If the TMG review of trial monitoring reports identifies issues that cannot be resolved through contact with sites by email or telephone, or if significant issues are identified, a triggered site visit may be indicated. Examples of triggers might include: identification of clear differences between expected and actual recruitment rates, no recruitment for an extended period at site, identification of outlying screening failure rate at a particular site; missing primary outcome data above a pre-defined acceptability threshold; a higher or lower SAE reporting level at site in comparison with the trial as a whole that cannot be reasonably explained by knowledge of patient characteristics; repeated violations in the timing of consent and trial related procedures, repeated non-receipt of consent forms within the timelines specified in the protocol or inaccuracies in the completion of consent documentation; repeated use of superseded versions of the Patient Information Sheet and Consent forms (PISC); repeated evidence of protocol deviations (such as recruitment of ineligible participants); and repeated discrepancies in the assignment of randomization number at a given site.

Following the triggered visit a site monitoring visit report will be completed detailing the reason for the visit, the processes reviewed, issues identified and discussed with the trial staff at the visit, as well as corrective measures to be implemented. The site monitoring visit report should be reviewed and signed by the monitor and PI on site and should be included in the trial monitoring reports for review by the TMG. A follow-up visit will be arranged with the site staff to ensure that any action points outlined in the site monitoring visit report have been acknowledged and rectified.

### The trial monitoring report

The TMG are responsible for reviewing monitoring activities undertaken during the trial and for authorising the appropriate subsequent action to be undertaken. These activities are summarized in a trial monitoring report which is prepared by the Trial Coordinator (TC) with input from other team members as appropriate, and is reviewed according to a regular schedule which should be pre-specified in conjunction with the risk assessment and detailed in the monitoring plan. Higher risk trials, or fast recruiting trials, may require more frequent reviews of trial monitoring reports, and it may not always be necessary to present all monitoring activities in every trial monitoring report. For example, a trial experiencing recruitment difficulties may require additional TMG review of recruitment patterns and screening failure rates. The trial monitoring report discussed at these meetings would not necessarily require all pre-planned monitoring activities to be summarized. The frequency of review of trial monitoring reports may also need to be amended during the trial if issues that require immediate action are identified.

The content of the trial monitoring reports will vary by trial but would generally include summary results of the central monitoring activities, statistical monitoring, issues raised from TSC and open IDSMC meetings, and on-site monitoring, with any relevant site monitoring visit reports included as an appendix to the report.

To ensure that there is a record of any decisions made by the TMG, the trial monitoring report includes an 'actions' section listing all actions requested as a result of TMG review. This list of actions would also be included in the subsequent trial monitoring report for review by the TMG to ensure that all previous actions have been carried out with a satisfactory outcome.

A site may be closed on the authority of the sponsor if they have not enrolled any participants for a considerable amount of time or the enrolment rate is not acceptable, or if the site is non-compliant with trial procedures or regulatory requirements. During review of the trial monitoring report, the TMG will consider whether any serious breaches in Good Clinical Practice have occurred which would be reported to the Sponsor who would be responsible for informing the MHRA.

Although there may be some overlap in content, this trial monitoring report should not be confused with reports which are prepared by the trial statistician and presented to the IDSMC. The IDSMC reports are usually for review only by the IDSMC since they often include results of analyses that compare treatments and these interim results could potentially introduce bias if released to the trial team.

## Conclusions

Clinical trials require measures to be taken to assure the quality of data, reliability of results, and to protect participants’ rights and safety. Recent developments in the literature by international bodies and regulatory agencies [3-6] have supported the need for risk-proportionate approaches to monitoring. The risk-proportionate approach adopted by the CTRC, an active, non-commercial UK clinical trials unit, is described in this paper to aid dissemination of methods, promote discussion and contribute to the evidence base that is currently lacking. However, further empirical evidence is required to thoroughly evaluate the costs, and advantages and disadvantages of alternative methods. Further, more in-depth statistical monitoring may be required for the detection of fraud [12], or to supplement simple approaches [2] if problems are highlighted. The use of statistical monitoring methods is an area of active research and appropriate use of these methods requires careful consideration of issues such as multiple testing and trial size.

Recommendations made by the CTTI project on effective and efficient monitoring as a component of quality highlight that ‘no single monitoring approach is appropriate or necessary in all circumstances’ , and that the ‘monitoring approach for a given clinical trial should be tailored to the needs of that trial and may combine several methods’ [3]. These points, along with other recommendations made by CTTI (many of which are reflected in the CTRC approach) should be kept in mind when developing the trial monitoring plan in conjunction with the trial risk assessment. Furthermore, by publishing the approach taken by CTRC we are supporting an ancillary recommendation made by the CTTI project, to ‘Share knowledge and experiences’ [3], and we fully encourage further sharing and discussion amongst the trials community so that best practices may be established.

## Abbreviations

ADR: adverse drug reaction; AE: adverse event; CI: Chief Investigator; CF: cystic fibrosis; CRF: Case Report Form; CTU: Clinical trials Unit; CTRC: Clinical Trials Research Centre; CTTI: Clinical Trials Transformation Initiative; CV: Curriculum Vitae; DH: Department of Health; DM: Data Manager; DMP: Data Management Plan; DSUR: Development Safety Update Report; EMA: European Medicines Agency; FDA: Food and Drug Administration; GCP: Good Clinical Practice; HTA: Health Technology Assessment; ICH: International Conference on Harmonization; IDSMC: Independent Data and Safety Monitoring Committee; IMP: investigational medicinal product; IS: information systems; IV: intravenous; MHRA: Medicines and Healthcare Products Regulatory Agency; MRC: Medical Research Council; PI: Principal Investigator; PISC: Patient Information Sheet and Consent; REC: Research Ethics Committee; RSA: Research Site Agreement; SAE: serious adverse event; SDV: source data verification; SmPC: summary of product characteristics; SMT: Senior Management Team; SOPs: Standard Operating Procedures; SUSAR: suspected unexpected serious adverse reaction; TC: Trial Coordinator; TM: Trial Monitor; TMF: Trial Master File; TMG: Trial Management Group; TMR: Trial Monitoring Report; TMS: Trial Management System; TORPEDO-CF: Trial of Optimal TheRapy for Pseudomonas EraDicatiOn in Cystic Fibrosis; TS: Trial Statistician; TSC: Trial Steering Committee; UKCRC: United Kingdom Clinical Research Collaboration.

## Competing interests

The authors declare that there are no competing interests.

## Authors’ contributions

CTS contributed to the development of the monitoring SOP which forms the basis of the paper and drafted the manuscript. PRW contributed to the development of the monitoring SOP which forms the basis of the paper and helped draft the manuscript. AJ, AS and SLH developed the TORPEDO-CF risk assessment and TORPEDO-CF monitoring plan and helped draft the manuscript. CG contributed to the development of the monitoring SOP which forms the basis of the paper, developed the TORPEDO-CF risk assessment and monitoring plan and helped draft the manuscript. All authors read and approved the final manuscript.

## Supplementary Material

Additional file 1 TORPEDO-CF: Risk assessment form. Click here for file

Additional file 2 TORPEDO-CF Monitoring plan. Click here for file

Additional file 3 TORPEDO-CF Protocol summary. Click here for file
