# Supplementary material for: Risk-proportionate clinical trial monitoring: an example approach from a non-commercial trials unit
Source: Trials. 2014 Apr 16;15:127. doi: 10.1186/1745-6215-15-127 (PMC4022377; doi:10.1186/1745-6215-15-127)
Supplement: Additional file 1 — TORPEDO-CF: Risk assessment form. [file 1745-6215-15-127-S1.docx]

-

| **Project Acronym:** | TORPEDO-CF | **Full Name of Project** | **Trial of Optimal TheRapy for Pseudomonas EraDicatiOn in Cystic Fibrosis** | |
| --- | --- | --- | --- | --- |
| **Chief Investigator (CI):** | Dr Simon Langton Hewer | **Employer of CI:** | Bristol Royal Children’s Hospital | |
| **Trial of IMP:** | Ceftazidime & Tobramycin Vs Ciprofloxacin | **Sponsor(s):** | University Hospitals Bristol NHS Foundation Trust | |
| **EudraCT N^o^ (if applicable)** | 2009-012575-10 | **Funder:** | HTA | |
| **ISRCTN (if applicable)** | ISRCTN02734162 | **Funder Ref:** | HTA 07/51/01 | |
|  | | | | |
| **Date of Assessment** | | **17^th^ August 2009** | | |
| **Number of Risk Categories Identified** | | **30** | | |
| **Range of scores per category (min-max)** | | **1-25** | | |
| **Minimum Possible Total Score**  **Maximum Possible Total Score**  (Sum maximum score possible i.e. 25 x number of categories) | | **25**  **750 (25 X 30)** | | |
| **Total Score for Trial**  (Sum of scores from all Categories) | | **146** | | |
| **Mean Score for Trial**  (Calculated as total score/number of categories) | | **4.86** | | |
| **Overall % Risk**  (Calculated as [Total score/maximum possible score] x 100) | | **19.46%** | | |
| **Category of Risk (circle)** | | Low | | If score ≤ 33% |
|  |  | Moderate | | If score ≥34 to ≤ 67% |
|  |  | High | | If score ≥ 68 to ≤ 100% |

| **Patient Hazards/ Research Staff Hazards (Rights and Safety)** | | | | | |
| --- | --- | --- | --- | --- | --- |
| **Generic Hazard** | **Impact if it happens (I)**  1 Low  2 Moderate  3 Significant  4 Severe  5 Catastrophic | **Likelihood of it happening (L)**  1 Remote  2 Unlikely  3 Possible  4 Likely  5 Certain | **Risk (R)**  IxL | **Specific Hazard** | **Management Strategies** |
| **Interventions**  Ciprofloxacin  Ceftazidime & Tobramycin | 1  (Justification: from Similar trials comparing Ciprofloxacin and colistin to other treatment regimens, expected AEs are not serious, leading to very low rates of withdrawal due to adverse events. Sample size calculation has taken into account participants that may withdraw from the trial due to Adverse Drug Reactions)  2  (Justification: expected AEs are not serious. Initial dose of medication given as inpatient to monitor for adverse drug reactions prior to home IV)  2  (Justification: As above)  2  (Justification: As above) | 4  4  (Justification: increased risk of ADR if IV treatment given at home without medical supervision)  4  (Justification: As above)  4  (Justification: As above) | 4  8  8  8 | Risk of adverse effects from trial drugs  Risk of adverse effects from trial drugs (Ceftazidime, Tobramycin)  Localised reactions to IV injection  Risk of Home IV administration | Selection of Principal Investigators (PIs) /sites who are experienced in dealing with Cystic Fibrosis (CF) patients and have experience with trial treatments  Trial regimens used in accordance with CF trust guidelines for the treatment of *P. aeruginosa* in CF patients  Management strategies in place to ensure appropriate and timely reporting of Serious Adverse Events (SAEs)   - Site Research Staff training on SAE and SUSAR reporting (staff includes PI, appropriate site staff Pharmacist, Microbiologist - Clinical Trials Unit (CTU) to ensure sites have full understanding of SAE reporting process - Circulation of an up to date Summary of Product Characteristics (SmPC) to participating sites with full profile of side effects - CTU protocol clearly defines roles and responsibilities for Pharmacovigilance and timelines of expedited reporting   SAE/ADR reporting to CTU according pharmacovigilance section of protocol  Oversight of safety reporting by Independent data and safety monitoring committee (IDSMC).  Clinical Trials Authorisation obtained from MHRA  Trial supplies produced to Good Manufacturing Practice and Good Laboratory Practice  Trial stickers on patient notes, according to local policy for alerts  As above  Home IV only given to participants that are deemed medically stable and have been trained in home IV usage  Home treatment only given to participants that have previous experience of home IV administration  Initial treatment given as inpatient where participant is closely monitored to measure for peak and trough levels of tobramycin  Serum creatinine and tobramycin serum concentrations measured before and during IV treatment |
| **Inexperienced Clinical team** | 3 (Justification: incorrect dosing could lead to ADR. Incorrect dosage could result ineffective treatment if dose to low or result in ADR if to high) | 1  (Justification: Trial treatments are being used in accordance with the CF trust guidelines for the eradication of *P. aeruginosa* in CF patients. All centres have experience of both treatment regimens. IV treatment given in accordance with local practice and only recommended to adult population with experience of home IV administration) | 3 | Incorrect advice to participants about taking trial medications   - risk of over dose leading to adverse effects - risk of under dose leading to sub-therapeutic dose - Incorrect Management of IV treatment - Home IV (IV treatment given in accordance with local practice and only recommended to adult population with experience of home IV administration) | Site research staff with significant level of delegated trial duties provided with training on GCP and (PI, appropriate site staff)  PIs/sites with experience in dealing with Cystic Fibrosis (CF) patients and have experience with trial treatments  Trial regimens used in accordance with CF trust guidelines for the treatment of *P. aeruginosa* in CF patients.  ceftazidime, tobramycin, ciprofloxacin & colistin compliance/ monitored by PI or designated team member at the end of 3 month treatment period, and recorded in CRF  Ciprofloxacin patient diary for recording how many tablets taken over 3 month course  Site staff delegation log with clearly defined delegation of responsibility |
|  | 3  3 | 1  (Justification: Similar trials have reported only a small number of participants that did not comply with protocol treatment regimen. Trial treatment being used in line with normal clinical practice)  1 | 3  3 | Protocol deviation  (non-compliance with treatment)  Incorrect eligibility assessment | Site delegation log Kept in site file. Each member of staff with delegated duties will sign log and this will be countersigned by PI  PI and appropriate personnel CVs from participating sites to ensure appropriate qualifications and suitability to conduct the study  Training for new PIs at site |
| **Consent**   - **uninformed** - **absent** - **pressured** | 3  3 | 1  (Justification: Eligibility criteria include ability to give written informed consent)  1 | 3  3 | Vulnerable paediatric patient population which could lead to uninformed assent /consent  Insufficient Time to allow participants to consider taking part in trial | Research Ethics Committee (REC) approved Patient Information Sheet and Consent (PISC) document notifying patients that a copy of the signed consent form will be sent and stored at CTU for central monitoring  Participants must have sufficient time to consider recruitment onto study. Recruiting centres will be provided with poster, leaflets and PISCs to display in CF clinics  Centres provided with posters and leaflets to advertise the trial to potential participants and give greater time for potential participants to consider their involvement in the study  The PI and all staff on delegation log with significant trial responsibilities to have GCP training and provide evidence of such to CTU prior to green light  Consent log kept in the site file which is faxed to CTU monthly |
|  | 4 | 1 | 4 | No evidence of consent | All staff on delegation log to have GCP training and protocol training highlighting consent process  Sites provided with screening logs to record all participants considered for the trial. This Log will be kept in the site file and will regularly be requested by the CTU to ensure compliance  Investigator and appropriate personnel provided with training; awareness that a copy of signed PISC also to be retained in patient notes. |
|  | 3 (Justification: Similar trials comparing Ciprofloxacin and colistin to other treatment regimens had very low rates of withdrawal) | 1  (Justification: Participating centres will advertise the trial by using REC approved posters, leaflets and PISC to raise awareness of the trial with potential participants and increase the time for the participant to consider the trial. Minimum time specified in protocol to allow for fully informed consent 24 hrs. Green light process requires GCP training for PI prior to opening a centre for recruitment) | 3 | Uninformed consent leading to withdrawals | Withdrawals logged in CRF  TC to check withdrawal rates |
|  | 3 | 1 | 3 | Incorrect Version of, or control, of PISC | CTU process in place for version control to include notification to site to mark old versions of documents as superseded, one copy to be stored in Site File with current documents for audit purposes  TC to ensure that patients re-consent using updated consent forms if substantial amendments are made to protocol which affect treatment or follow up  Site staff will be trained in trial procedures and the importance of using the correct PISC. |
| **Patient Confidentiality** | 3 | 2  (Justification staff trained on  importance of patient confidentiality) | 6 | Breach of confidentiality | Anonymisation of all trial data apart from consent form which is stored at CTU in secure storage area and not databased. Logs maintained at sites to track patients locally.  Training of site research staff on importance of patient confidentiality  CRFs do not collect patient names  REC approved processes  Secure storage facilities within the CTRC to store CRFs and Consent forms  CTU staff Trained in principles of Data Protection Act 1998 |

| **Study Hazards (Completion and Reliability)** | | | | | |
| --- | --- | --- | --- | --- | --- |
| **Generic Hazard** | **Impact if it happens (I)**  1 Low  2 Moderate  3 Significant  4 Severe  5 Catastrophic | **Likelihood of it happening (L)**  1 Remote  2 Unlikely  3 Possible  4 Likely  5 Certain | **Risk (R)**  IxL | **Specific Hazard** | **Management Strategies** |
| **Organisational complexity** | 3  4  2  3  2  3 | 3  (Justification: CTU has management strategies in place to minimise risk)  1  (Justification: Random-isation not possible without Green Light status)  2  2  2  1  (Justification: Participating centres provided with training on pharmacovigilance process which is clearly defined in the protocol[section 9.5] and listed in the delegated responsibilities of the RSA) | 9  4  4  6  4  3 | Multi-centre study which can lead to   - Inconsistencies across sites in CRF completion - Inconsistencies across sites in SAE reporting - Communication problems - Problems disseminating updated trial documentation (version control) - Recruitment Problems   Green light process not followed:   - No Local REC, - No Research &Development approval - No Research Site Agreement for participating centres - No site initiation   Lack of communication (internal):   - New version of documents not distributed to all sites - Awareness of site issues not communicated   Lack of communication (external):   - Protocol violations - Incorrect forms used in study - Breaches in confidentiality - TMF contain incorrect documents   Insufficient training at site:   - Non-compliance with protocol - New staff not captured in training regimen   Management of SAE’s not followed in accordance with protocol: Drug safety issues not identified at an early stage | Training of all site research staff (initiation, GCP, SAE reporting etc.)  CI to review and assess SAEs  Central trial co-ordinator to manage communication and to be first point of contact for all sites to ensure consistency across sites  Standardised paper Case Report Forms (CRFs) with CRF completion guidelines  CTU to regularly check status of; missing forms, missing data items, discrepant data (batch validations) and completeness of data  Procedures set in place to notify relevant parties of serious breaches within the trial  Trial advertisement, regular newsletters  Principal investigator to sign declaration that they are working to the current version of the protocol  Agreements/contracts in place with clear delegation of responsibilities  Green Light Process in place to ensure recruiting centres are not opened until appropriate paperwork is in place and that site initiation has taken place  Sites only opened once all appropriate paperwork has been received from sites, and site initiation has occurred. This process is defined in CTU SOP (TM017: Study Initiation at CTRC) and CTU staff trained prior to opening recruiting centres (TM018: Study initiation at Site)  Barriers built into web randomisation process to prevent participants being recruited until green light sign off  TC for the trial will be the only one to review documentation prior to green light sign off  TC to send notification to recruiting centres once they are deemed open to recruitment  Sites provided with regular updates on their progress and sent copies of missing CRF requests and data clarification forms as detailed in the trial specific Data Management Plan  Trial Management System (TMS) containing communication with sites to allow for trial cross cover and consistency in the management for trial such there be a change in CTU staff  Protocol violation log kept by TC  Document management system to ensure latest version being used at site  Training site research staff  Continual site training throughout the project  New site staff sign delegation log and submit CV to CTU  Pharmacovigilance process clearly detailed in trial protocol  SAE training given to participating centres as part of site as part of initiation |
| **Study Power**  **Recruitment**  **Consent** | 3  (Justification: Current Evidence base contains 4 small RCTs of generally poor methodological quality) | 3  (recruitment schedules developed with information from HTA funded feasibility study) | 9 | Inadequate recruitment leading to insufficient study power | Statistical input to design and power calculation  Realistic recruitment schedules developed with information from HTA funded feasibility study  Adequate resources e.g. ample number of sites  Trial promoted at relevant Respiratory / CF conferences and meetings to encourage centres to take part  Sites recruitment schedule confirmed at initiation and reviewed/discussed as necessary  Trial newsletter sent to sites on a quarterly basis to keep recruitment momentum and motivate centres with low recruitment figures |
| **Study Results** | 2  2  3 | 2  2  (Justification: designated research staff at participating centre given training in web randomisation process, and issued with individual login details)  2 (Justification: there are no financial incentives provided to individual PIs by the trial) | 4  4  6 | Violation of eligibility criteria  Incorrect randomisations  Fraudulent Data | Ensure site staff are trained on the protocol and using up to date versions of the protocol  CTU staff to review eligibility criteria checklist and other data on screening/randomisation CRF  As above  CTU office cover during working hours to provide technical advice for sites having problems with randomisation process  TC to keep report of randomisation errors and notify statistician as appropriate  Hospital trusts provided with per patient payments to cover for the additional time needed for data collection and administration. Detailed in the RSAs |
|  | 3 | 3  (Justification: outcomes are routinely collected as part of normal clinical care) | 9 | Missing primary andsecondary outcome results | Staff training on CRF completion and importance of minimising missing data  Regular reports of missing data by TC/statistician for review by TMG  Data management plan to be followed |
| **Staff competence and experience** | 2  2 | 2  2 | 4  4 | Data collection paper CRF completion incorrect or incomplete  Study procedures not followed according to protocol | Training:   - CTU personnel - Site personnel (at initiation and also supply sites with CRF completion guidelines)   MACRO validation checks |

| **Organisational Hazards** | | | | | |
| --- | --- | --- | --- | --- | --- |
| **Generic Hazard** | **Impact if it happens (I)**  1 Low  2 Moderate  3 Significant  4 Severe  5 Catastrophic | **Likelihood of it happening (L)**  1 Remote  2 Unlikely  3 Possible  4 Likely  5 Certain | **Risk (R)**  IxL | **Specific Hazard** | **Management Strategies** |
| **Additional tests / non-clinical test results** | 3 | 3 (genotyping of samples links directly to the primary outcome of the trial, and is not usual practice for confirming infection. Microbiology staff will be made aware of the trial and high visibility labels will be used on all potential participant sputum /cough sample to highlight the importance of storing the sample. Microbiology labs will be provided with consumables and instructions) | 9 | Genotyping samples not kept and sent to the Health Protection Agency for genotyping | High visibility labels used to identify cough / sputum samples are being collected for use in this study  Clear instructions in the protocol relating to collection of samples for genotyping.  Microbiology staff invited to attend participating centre initiation meetings.  Participating centres provided with Postage kits, and consumables |
| **Liability** | 4 | 1 | 4 | Legal obligations under UK Regulations for clinical trials involving medicinal product | Sponsor clearly identified and clarity of liability arrangement with collaborators  REC approved PISC with clear liability information (e.g. liability for negligent harm)  Ongoing training program for all staff to include ICH GCP  Ensure all sites have the SmPC for all medicinal products used during the trial |
| **Service impact** | 2 | 1 (Justification: Trial procedures part of normal clinical care, study visits in line with normal clinic visits as much as possible) | 2 | Disruption to normal clinical care | Participating centres receive payment to reimburse for time taken for data collection & administration |
| **Intellectual Property** | 2 | 1 | 2 | Potential opportunities overlooked or lost opportunity due to disclosure of results | Contracts in place detailing Intellectual Property rights  Confidentiality agreements |

**Risk Assessment Matrix**

|  | **1 Remote** | **2 Unlikely** | **3 Possible** | **4 Likely** | **5 Certain** |
| --- | --- | --- | --- | --- | --- |
| **1 Low** | 1 | 2 | 3 | 4 | 5 |
| **2 Moderate** | 2 | 4 | 6 | 8 | 10 |
| **3 Significant** | 3 | 6 | 9 | 12 | 15 |
| **4 Severe** | 4 | 8 | 12 | 16 | 20 |
| **5 Catastrophic** | 5 | 10 | 15 | 20 | 25 |

**Risk Management Key**

| **Action and time scales** |
| --- |
| Immediate action must be taken to manage the risk. Control measures should be put in place which will have the effect of reducing the impact of an event or the likelihood of an event occurring. A number of control measures may be required. |
| Significant resources may have to be allocated to reduce the risk. Where the risk involves work in progress urgent action should be taken. |
| Efforts should be made to reduce the risk, but the costs of prevention should be carefully measured and weighed against the impact of the event. Establish more precisely the likelihood of harm as a basis for determining the need for improved control measures. |
| On or below this level a risk is acceptable. Existing controls should be monitored and adjusted. No further action or additional costs are required. Consideration may be given to a more cost-effective solution or improvement that imposes no additional cost burden. |
| Acceptable risk. No further action or additional controls are required. Risks at this level should be monitored, and reassessed at appropriate intervals. |

This TORPEDO-CF Risk Assessment Version 2.0 has been completed and approved by the following personnel

**On behalf of Sponsor:**

**Print Name:** _________________________________ **Date (dd/mm/yyyy)** _________

**Signature:** _________________________________

**CTRC Director**

**Print Name:** _________________________________ **Date (dd/mm/yyyy)** _________

**Signature:** _________________________________

**Chief Investigator**

**Print Name:** _________________________________ **Date (dd/mm/yyyy)** _________

**Signature:** _________________________________
