# Supplementary material for: Risk-proportionate clinical trial monitoring: an example approach from a non-commercial trials unit
Source: Trials. 2014 Apr 16;15:127. doi: 10.1186/1745-6215-15-127 (PMC4022377; doi:10.1186/1745-6215-15-127)
Supplement: Additional file 2 — TORPEDO-CF Monitoring plan. [file 1745-6215-15-127-S2.docx]

| Appendix 2. TORPEDO-CF MONITORING PLAN |
| --- |

| **Project**  **Acronym:** | TORPEDO-CF | **Full Name of Project** | | **T**rial of **O**ptimal The**R**apy for **P**seudomonas **E**ra**D**icati**O**n in **C**ystic **F**ibrosis |
| --- | --- | --- | --- | --- |
| **Chief Investigator (CI):** | Dr Simon Langton Hewer | **Employer of CI:** | | Bristol Royal Children’s Hospital |
| **Trial of IMP:** | Ceftazidime and Tobramycin Vs Ciprofloxacin | **Sponsor(s):** | | University Hospitals Bristol NHS Foundation Trust |
| **EudraCT N^o^**  **(if applicable)** | 2009-012575-10 | **Funder:** | | HTA |
| **ISRCTN**  **(if applicable)** | ISRCTN02734162 | **Funder Ref:** | | HTA 07/51/01 |
|  | | | | |
| **Version number and date** | | | **1.0 : 1^st^ June 2012** | |
| **Superseded version number and date** | | | **Not Applicable** | |
| **Author(s)** | | |  | |
| **Overall % Risk** (As per risk assessment) | | | **19.46%** | |
| **Category of Risk** (As per risk assessment) | | | **** Low **** | |

|  |  |
| --- | --- |

Contents

[3. SUMMARY OF MONITORING PLAN](#_Toc326914008)

[4. TRIAL OVERSIGHT COMMITTEES](#_Toc326914009)

[5. PROTOCOL DEVIATIONS](#_Toc326914013)

[6. GENERAL MONITORING ACTIVITIES](#_Toc326914014)

6.[1.CENTRAL MONITORING ACTIVITIES](#_Toc326914015)

6.[2. SOURCE DOCUMENTATION AND SDV PLAN](#_Toc326914016)

6.[3. TRIAL CLOSE-OUT](#_Toc326914017)

[7. TRIAL MONITORING PLAN SIGNATURES](#_Toc326914018)

# SUMMARY OF MONITORING PLAN

This monitoring plan determines the nature and extent of monitoring required for the TORPEDO-CF trial, and has been developed taking into consideration the requirements of the trial and the outcome of the risk assessment. Monitoring of the TORPEDO-CF trial will include (i) restricting trial and individual site opening until the green-light process has been followed to ensure correct documentation and regulatory compliance (SOP TM017 Study Initiation in CTU, SOP TM018 Study Initiation at Site), (ii) central monitoring with triggered on-site monitoring if any persistent or serious concerns are identified by central monitoring (if deemed appropriate following discussion by the trial management group (TMG)), and (iii) routine on-site close-out visit to each site.

Central monitoring in the TORPEDO-CF trial includes central review of all consent forms, monitoring of recruitment, protocol deviations, adverse events, missing primary outcome data, visit schedules, and quality and timeliness of case report form (CRF) completion, return, data entry and review of data entry.

Monitoring activities may be reported in any or all of the CTRC Head of Section Meetings (held monthly), in TMR presented to the TMG (every month), in IDSMC reports (at least annually). The frequency of these meetings may be adapted as necessary. When an IDSMC report coincides with the TMR the IDSMC report will replace the TMR.

The trial statistics team will be responsible for producing the IDSMC report. The Trial co-ordinator and data manager will be responsible for producing the Head of section meeting reports, trial monitoring reports and for undertaking monitoring activities unless otherwise stated. The trial statistics team and the IS team will be consulted to help with extracting reports and producing appropriate summaries of data.

The Trial Steering Committee (TSC) and Independent Data and Safety Monitoring Committee (IDSMC) also play a role in the monitoring of the TORPEDO-CF trial. The TSC and IDSMC charters provide further detail on the role of these oversight committees in safeguarding the interests of participating patients and monitoring the conduct of the trial.

# TRIAL OVERSIGHT COMMITTEES

Membership of the TORPEDO-CF Trial Oversight Committees are listed within the Oversight Committee Membership Document located within the TMF.

Trial Steering Committee (TSC)

The role of the TSC is to provide overall supervision for the trial and provide advice to the funder and the sponsor through its independent chairperson. The ultimate decision for the continuation of the trial lies with the TSC. Further details are provided in the protocol and TSC Terms of Reference.

Independent Data and Safety Monitoring Committee (IDSMC)

The IDSMC will be responsible for reviewing and assessing recruitment, interim monitoring of safety and effectiveness, trial conduct and external data. The IDSMC will provide a written recommendation to the Trial Steering Committee concerning the continuation of the study. Further details are provided in the protocol and IDSMC Charter.

Trial Management Group (TMG)

The TMG will be responsible for the day-to-day running and management of the trial and will aim to meet/teleconference monthly. The TMG will be responsible for the review of trial monitoring reports and recommending arising actions.

# PROTOCOL DEVIATIONS

The following table lists potential deviations of important protocol specifications, including eligibility criteria, treatment regimens and study assessments. The occurrence of these deviations will be monitored during central monitoring using the approach listed in the table.

**Note:**

1. **Impact** refers to the impact of the potential protocol deviation on the **risk of introducing bias** in the trial results, increasing the risk of harm to the participant as a result of their condition or its treatment or a violation of patient rights.

Any increased risk of harm or violation of patient rights will be graded as major. For the risk of introducing bias, impact will be graded as:

- **major** (in which case patients who experience this protocol deviation would be **excluded** from the “per protocol” analysis set)
- **minor** (in which case patients who experience this protocol deviation would be **included** in the “per protocol” analysis set)

2. **Justification** refers to the protocol-specific justification for the assessment of the impact of each potential protocol deviation. Justification may relate to statistical concerns of bias, generalisability, loss of power or to participant safety in reference to the inherent risks in standard medical care or patient rights and well being.

All protocol deviations of the listed protocol specifications will be included in the trial monitoring reports and IDSMC reports and presented overall and by site when numbers recruited per site are sufficient. The TS should prepare this information.

| **Protocol specification** | **Potential deviation(s)** | **Impact** | **Justification** |
| --- | --- | --- | --- |
| ***Inclusion criteria*** |  |  |  |
| Children over the age of 28 days (no upper age limit) | Outside of age range | ***Minor*** | Statistical concerns- impact on generalisability  Impact on safety not greater than standard medical care.  Drugs have been used off label extensively. Deviation outside of trial limits would not invoke risk of patient harm beyond that seen in standard medical care where similar doses are used |
| Consent | Consent not obtained | ***Major*** | Violation of participant rights |
| Participant able to start treatment no later than 21 days from the date of *P.aeruginosa* positive microbiology report | A patient starting treatment after 21 days. | ***Minor*** | Statistical concerns – if related to treatment arm requiring a hospital stay then this could introduce bias.  Time to start treatment is reported by treatment arm in the closed section of the IDSMC overall in the TMR and open section of the IDSMC.  Treatment would not be withheld from patient but patients own circumstances may impact on time to initiate treatment. Therefore no greater risk to safety than that observed in standard medical care |
| Patient should be either *P.aeruginosa* naive or free | Inclusion of patient who has not been clear of P.aerginosa for 12 months. | ***Minor*** | Treatment may be altered for chronic infections. Patient would not be harmed by trial regimens but may not be optimal therapy therefore minor impact on safety |
| ***Exclusion criteria*** |  |  |  |
| Previous randomisation in TORPEDO-CF | Patient could receive therapy that has previously not worked | ***Major*** | Statistical concern- non independence of patients impacting power  Impact on safety |
| ***Treatment regimen*** |  |  |  |
| *Compliance* | Use of non-specified protocol dosing regimen | ***Major*** | Impact on generalisability of trial results as well as potential safety |
| *Withdrawal from treatment due to safety* | Premature discontinuation of randomised treatment | ***Major*** | Risk of bias if withdrawal rates differ by trial arm but patient would still be followed up with primary outcome measured consent allowing- major as would impact on interpretation of primary outcome. Overall withdrawal due to safety monitored in open IDSMC and TMR and by treatment arm in closed IDSMC  Known safety profiles in both groups and risk no greater than that in standard medical care- minor |
| *Withdrawal from treatment due to patient preference* | Premature discontinuation or refusal to commence randomised treatment | ***Major*** | Bias – choice of treatment non-randomised  Overall withdrawal due to safety monitored in open IDSMC and TMR and by treatment arm in closed IDSMC  No safety implications |
| ***Study assessments*** |  |  |  |
| Scheduled visits at 6,9,12,18,21 and 24 months | Missing or Visits occur outside seven day window either side of visit date | ***Minor*** | No risk of bias-Not required for primary outcome  Safety -minor as in practice treatment completed and risk of impact of missed visits not increased beyond routine practice |
| Scheduled visit at 3 month and 15 month | Missing or Visits occur outside 14 days of treatment and cough / sputum samples should be taken 48 hours after cessation of eradication treatment. | ***Major*** | Statistical concerns– loss of power and potential for bias. Data from both time points required for primary outcome, could impact generalisability and power.  Attendance at both visits monitored overall in open IDSMC and TMR and by treatment arm in closed IDSMC.  Safety -minor as in practice treatment is completed and risk of impact of missed visits not increased beyond routine practice |

# General monitoring activities

General monitoring activities are identified within the relevant CTRC SOP and aim to ensure compliance with study protocol and regulations.

Trial coordinator to monitor site progress and problems and report issues within TMR

| **Generic Hazard identified in risk assessment** | **Central monitoring activity** | **CTRC SOP or checklist** | **Information to be considered for reporting** |
| --- | --- | --- | --- |
| Interventions | - CTRC green light process for regulatory approval - Green Light Process at site to ensure approvals and contracts in place prior to site initiation - Production of annual safety report and DSUR | CTRC Green-light checklist  SOP TM026 | - Indicate progress/timeframes to achieve approvals for amendments - Summary of progress for each site to reach initiation and progress of amendments through local Research & Development departments |
| Inexperienced clinical team | - Verify PI and appropriate personnel have undertaken trial specific training at Green-light checklist. | Green Light Initiation Checklist-External | - Cross-check signature and delegation log against CRF usernames/signatures - Request evidence of GCP training for new staff added to delegation log |
| Consent | - PI and appropriate staff have undertaken trial specific training at Green-light checklist. - Up to date version of PISC forms are used at site via document control process - 100% review of all consent forms which will include checking version number of PISC issued - Patients re-consented using updated consent forms if relevant | - Site visit log for Initiation - Signed delegation log - Consent form checklist - SOP GE007 Management of Serious Breaches of GCP or the Protocol | Issues with consent process or its documentation by site presented to the TMG |
| Patient Confidentiality | - Approvals in place prior to site initiation and Green light sign-off - Verify PI and appropriate personnel have undertaken trial specific training - CRFs and patient diaries checked to ensure anonymised apart from PISC | - SOP TM018 - Green Light Initiation Checklist - SOP DM004 Data Protection - TORPEDO-CF approved Data Management Plan | Issues in sites sending patient identifiable data – report on actions taken and response where persistent (see Data Management Plan) |
| Organisational complexity | - Centralised receipt of standardised paper CRFs. - Internal audit of TMF to check green light process is being followed - Continually cross-check of new staff | - Protocol - SOP GE004 | Request CV and evidence of GCP for new staff added to delegation log. |
| Study results | - Site research staff have attended specific training - Maintain a deviation log - Randomisation errors are monitored | - Site visit log for Initiation - Signed delegation log | - Request CV and evidence of GCP for new staff added to delegation log. - Summary of difficulties encountered with randomising patients |
| Staff competence and expertise | - Training records at CTU - Trial staff have attended trial specific training | - SOP GE002 Maintenance of Training and Competency Records - SOP GE003 Staff Training - Site visit log for Initiation - Signed delegation log | Training records (CTU) |
| Liability | - Research site agreements and material transfer agreements are in place prior to trial initiation. - Independent oversight committees - Acknowledgement form sites that they have received SmPC | - SOP TM024 Clinical Trial Agreements/ Contracts - SOP GE006 Material Transfer Agreement - SOP TM004 Oversight by Trial Committees - SOP TM030 Approval and Management of Reference documentation within a Clinical Trial of IMP | Document tracking for issues of SmPC updates. |
| Intellectual Property | Contracts in place  Confidentiality agreements | - SOP TM024 Clinical Trial Agreements/ Contracts - SOP DM004 Data Protection | Report on end dates of contracts with CTU  Issues with breach of confidentiality |

| **Systems** |  |
| --- | --- |
| Validations within study database | - Automated checks to ensure data validity - Check that subject identifiers, data entry and dates are consistent on each CRF. Validation checks on MACRO are summarised in the IS file and at <https://ctrc.liv.ac.uk/crfonlineannotation/Home/Login> |
| Validations in web-based randomisation | - Checks to ensure patient eligibility |
| Communication between web randomisation system and study database for eligibility | - Randomisation number, date and treatment allocation entered on to web-based randomisation system is imported to study database automatically |

##

7.1. CENTRAL MONITORING ACTIVITIES

In general the following three stage process will be initiated if indicated:

1. Contact site to discuss issues

2. Provide additional remote training

3. Site visit may be necessary if problem persists despite appropriate action.

| ***Patient Recruitment*** | **Purpose: To ensure**   1. Trial recruitment is on target 2. Identify sites underperforming to identify issues 3. Identifying sites over recruiting for issues around resource and possible fraud | | | | | | | | | | | |
| --- | --- | --- | --- | --- | --- | --- | --- | --- | --- | --- | --- | --- |
| **What/How** | | | | **Who** | | **When/Where reported** | **Possible indication of problem** | | | | **Possible action if problem identified** | |
| Screening logs overall and by site  - Frequency of ineligible patients with summary of reasons  - Frequency of non-consenting of otherwise eligible patients with summary of reasons  -Summary of consent rate  - Patients eligible / patients screened  Graphical summary overall of:  - actual number of randomised participants (cumulative monthly total)  - expected number of randomised participants (cumulative monthly total)  - number of sites open and expected number of sites open on overall graph  Display of recruitment rates required to achieve target from date of site opening and at current month, revised recruitment rate for sites delayed to opening. | | | | Trial statistics team to lead on production  Involvement of TC and IS as appropriate  Trials stats team | | TMR  IDSMC  TSC  Also reviewed at monthly TMG meetings in between formal TMR | Identification of clear differences in recruitment rates between sites (unless otherwise expected) or no recruitment at a site for an extended period despite attempts to resolve by discussion with site staff.  Delays in site openings due to staffing resources.  Delays in site openings due to site specific protocol queries. | | | | 3 stage process | |
| ***Informed consent*** | Purpose: To ensure   1. Consent is obtained 2. Consent returned to CTU in the 7 day time frame specified in protocol | | | | | | | | | | | |
| ***What/How*** | | | | **Who** | | **When/Where reported** | **Possible indication of problem** | | | | **Possible action if problem identified** | |
| - Number and percentage of consent forms submitted outside the 7 day time frame overall and across sites. - Number and percentage of overdue consent forms not yet received at CTRC overall and by site - TC to flag persistent problems identified with sites from consent form checklist. | | | | TC and TS | | IDSMC TSC TMR | Non-receipt of consent forms  Repeated delays submitting consent forms to CTRC | | | | 3 stage process  Potential serious breach if consent forms are not received. | |
| ***Eligibility*** | Purpose: To ensure   1. Patients randomised are eligible | | |  | |  |  | | | |  | |
| Eligibility violations overall and across sites | | | | Statistics and IS | | See protocol deviations table | Repeated evidence of protocol violations; recruitment of ineligible patients. | | | | 3 stage process. | |
| ***Randomisation*** | Purpose: To   1. Identify problems with randomisation system 2. Ensure information entered in to web-based system is accurate 3. Ensure balance between trial arms for number of randomised patients | | | | | | | | | | | |
| ***What/ How*** | | | | **Who** | | **When/Where reported** | **Possible indication of problem** | | | | **Possible action if problem identified** | |
| - Missing randomisation numbers - Randomisation numbers out of sequence with date/time of randomisation - Allocations provided to site by web-based system match those within the lists provided by stats - Balance in patient numbers across trial arms | | | | Stats | | TMR  IDSMC  TSC  Closed IDSMC | Repeated discrepancies in the assignment of randomisation number at a given site  Randomisation number missed out  Unexpected imbalance in patient numbers | | | | If errors detected, randomisation system should be investigated. | |
| ***CRF Completion*** | Purpose: To ensure  1) CRFs are received and entered in a timely manner  2) CRFs are checked for missing data  3) data queries are responded to | | | | | | | | | | | |
| ***What/ How*** | | | | **Who** | | **When/Where reported** | **Possible indication of problem** | | | | **Possible action if problem identified** | |
| - CRFs expected versus actual received at CTRC by site by timepoint and overall. - Number of weeks CRFs overdue by CRF and by site. - Number of expected visits confirmed as missed by site. - CRFs entered compared to received at CTRC. - Data queries raised, resolved by site and overall by patient volume and length of follow up (eg per patient months) | | | | DM | | TMR | Quality of completion of the CRFs is persistently inadequate  CRFs persistently not sent to CTRC in a timely manner  Unusually high levels of missing CRFs/visits.  Data queries not resolved | | | | 3 stage process. | |
| **Primary Outcome** | Purpose: To ensure  1) Ensure accuracy of reporting of primary outcome  2) Ensure completeness of reporting | | | | | | | | | | | |
| Percentage of patients at site and overall with missing primary outcome variable  Number of patients with data discrepancies raised against the primary outcome in each state (open, responded, closed).  (See protocol deviations table) | | | | DM  Database specification agreed by statistics team/TC/IS | | IDSMC &TSC report | Site persistently submitting CRFs with missing primary outcome data – check against acceptability threshold of 5%  Site persistently submitting inconsistent/incorrect primary outcome data | | | | 1. Contact site immediately and stress the importance of complete and accurate primary outcome data 2. Site-visit to discuss with PI if problem persists, to include SDV of Primary Ooutcome data | |
| ***IMP / other trial material*** | | Purpose: To ensure  1) Ensure trial treatment protocol is being adhered to  2) Ensure participants are compliant with taking trial medication | | | | | | | | | | |
| ***What/ How*** | | | | **Who** | | **When/Where reported** | **Possible indication of problem** | | | | **Possible action if problem identified** | |
| IV –- measure for trough levels of tobramycin  IV – serum creatinine and tobramycin serum concentrations measured before IV treatment  Check of treatment diaries to check for compliance with protocol | | | | Stats | | TMR  TMR  TMR & IDSMC report | Recorded dose levels that are under or over protocol specifications.  Missing information on treatment diaries or missing treatment diaries. | | | | *3 Stage process* | |
| ***Laboratories (if applicable)*** | | Purpose: To   1. Ensure laboratories are sending data for primary outcome on pseudomonas Aeruginosa 2. Check that laboratories are keeping samples for genotyping | | | | | | | | | | |
| ***What/ How*** | | | **Who** | | | **When/Where reported** | | **Possible indication of problem** | | | **Possible action if problem identified** | |
| Regulator sample collection updates generated by health protection agency (HPA) to monitor the collection of samples and highlight any problems with centres not carrying out these procedures. | | | | TC | IDSMC | | | Lack of reports or incomplete laboratory data | | Contact lab to discuss trial requirements and training needs | | |
| ***Adverse Events*** | | Purpose: To ensure  1) safety data are reported accurately and completely  2) follow up information on AEs is obtained | | | | | | | | | | |
| ***What / How*** | | | | **Who** | | **When/Where reported** | **Possible indication of problem** | | **Possible action if problem identified** | | | |
| On receipt of paper CRFs check for missing or inconsistent data on related AE / SAE pages.  Obtain any follow up information required for ongoing related AEs / SAE or obtain confirmation that the AE is considered chronic or stable.  Summarise:  1) Rates of events across sites and in total  2) Summary of SAE/SUSARs  3) Adverse events by severity and relationship to study drug  4) Time delay submitting AE data and SAE data (site should submit SAE form within 24 hours of becoming aware of the event). | | | | DM to review completeness of AE CRF and follow-up of ongoing AEs.  DM/Stats to produce summary of AE and SAEs  IDSMC to review AE/SAE by treatment group and TSC review overall levels of AEs/SAES | | TMR & IDSMC report | Persistent delays submitting SAE form  Persistent inaccuracies/missing data on AE or SAE form | | | | 3 stage process. | |
| ***Data Correction/ Clarification*** | | Purpose: To ensure  1) data entered onto database is valid and complete  2) data queries are resolved in a timely manner | | | | | | | | | | |
| ***What / How*** | | | | **Who** | | **When/Where reported** | **Possible indication of problem** | | | | **Possible action if problem identified** | |
| **Summarise:**   - Total number of data discrepancies by site - % of the total number of discrepancies in open, responded, and closed states by site. - Time taken to close queries and the number of queries open or responded 4 weeks or more since the query was raised | | | | DM to use default MACRO data reports to produce summary. | | TMR | Persistent delays or inaccuracies in Data Clarification Forms  Persistent delays with resolution of data queries | | | | 3 stage process. | |
| ***CTRC database review*** | | Purpose: To ensure  1) data entered onto database is consistent with CRF | | | | | | | | | | |
| ***What / How*** | | | | **Who** | | **When/Where reported** | **Possible indication of problem** | | | | | **Possible action if problem identified** |
| Independent DM/TC to cross-check (CRF vs database)   - 100% of primary outcome data - 100% of randomisation data - Check of secondary data for first randomised patient at site and then every fifth patient.   Summarise percentage of data items with discrepancy and present in TMR. | | | | Independent TC/DM | | Head of Section Meetings / TMR | Error rate > 0.5% for key data items and > 3% error rate for other data items | | | | | 1. Correct any errors identified and check MACRO to identify if data entry errors are occurring due to problems with user acceptability for particular fields / forms. 2. Provide further training to DM if exceeds thresholds indicated 3. Check further random sample of CRFs |

7.2. SOURCE DOCUMENTATION AND SDV PLAN

Document defining Source data is located within the TMF. Each site signs a paper copy detailing the location of source data at site. This is received at the CTRC and stored in the TMF.

There is no planned SDV.

7.3. TRIAL CLOSE-OUT

Site closure and Trial closure are detailed in SOP TM035. Progress for each site on the checklist associated with the SOP will be summarised and presented within Head of Section meetings and TMRs.

# TRIAL MONITORING PLAN SIGNATURES

The TORPEDO-CF Monitoring Plan (version 1.0) has been approved by the following personnel:

**Trial Statistician**

**Name**

**Signature**

**Date (dd/mm/yyyy)**

**Senior Statistician or Head of Statistics**

**Name**

**Signature**

**Date (dd/mm/yyyy)**

**Senior Data Manager**

**Name**

**Signature**

**Date (dd/mm/yyyy)**

**Chief Investigator**

**Name**

**Signature**

**Date (dd/mm/yyyy)**

**Trial Coordinator**

**Name**

**Signature**

**Date (dd/mm/yyyy)**

**CTRC Director**

**Name**

**Signature**

**Date (dd/mm/yyyy)**
