# Supplementary material for: Risk-proportionate clinical trial monitoring: an example approach from a non-commercial trials unit
Source: Trials. 2014 Apr 16;15:127. doi: 10.1186/1745-6215-15-127 (PMC4022377; doi:10.1186/1745-6215-15-127)
Supplement: Additional file 3 — TORPEDO-CF Protocol summary. [file 1745-6215-15-127-S3.docx]

# APPENDIX 3. TORPEDO-CF Protocol summary

| Study Drugs: | Ceftazidime, Tobramycin, Ciprofloxacin, Colistin |
| --- | --- |
| Title of Study / Protocol Number: | Trial of Optimal Therapy for Pseudomonas Eradication in Cystic Fibrosis |
| Chief Investigator (s): | Simon Langton Hewer |
| Number of Study Centres and Distribution | CF centres throughout the UK. |
| Study Period: | 5 years |
| Main Objective(s): | This trial will assess whether fourteen days intravenous ceftazidime with tobramycin is superior to three months oral ciprofloxacin.  Both treatment regimens will be in conjunction with three months nebulised colistin. |
| Study Design: | Multi-centre, parallel group, randomised controlled trial comparing fourteen days of intravenous therapy to three months of oral therapy. |
| Number of Patients to be enrolled: | 280 are to be randomised in total, 140 in each arm of the study |
| Main criteria for inclusion: | 1. Diagnosis of CF 2. Children over the age of 28 days, older children and adult CF participants are all eligible with no upper age limitation. 3. Competent adults should provide fully informed written consent to participate in the trial. 4. Minors should have proxy consent by the parent or legal guardian and should provide assent where applicable to participate in the trial. 5. The participant should have isolated *P. aeruginosa* and should be either: 6. *P. aeruginosa –*naïve (i.e. has never previously isolated *P. aeruginosa*) or 7. *P. aeruginosa* –free (i.e. a minimum number of four consecutive *cough or sputum samples* should be *P. aeruginosa* free within a 12 month period to satisfy eligibility.) 8. The participant should be able to commence treatment no later than 21 days from the date of a positive P. aeruginosa biology report. |
| Main criteria for exclusion: | 1. Antibiotic resistance of the current *P. aeruginosa* sample to any of: ciprofloxacin, ceftazidime, tobramycin or colistin reported by local microbiology laboratory. 2. Known participant hypersensitivity to either ciprofloxacin, ceftazidime, tobramycin or colistin. 3. Other known contraindications to any of ciprofloxacin, ceftazidime, tobramycin or colistin including previous aminoglycoside hearing or renal damage. 4. Participant receiving *P. aeruginosa* suppressing treatment, in particular nebulised colistin or tobramycin, or oral ciprofloxacin for the previous 9 months. Please note, short courses of oral ciprofloxacin or intravenous antibiotics (with an anti-pseudomonal spectrum of action) are not an exclusion unless they are given to treat proven infections with *P. aeruginosa.* 5. Treatment with other anti-pseudomonal nebuliser. 6. Pregnant and nursing mothers (women of child bearing age will be counselled on the risks of becoming pregnant during the trial and will be offered a pregnancy test) 7. Previous randomisation in TORPEDO-CF study 8. Previous participation in another intervention trial within four weeks of taking part in TORPEDO-CF |
| Duration of Treatment: | Total maximum study duration for each participant is 24 months.  Participants will be seen every three months during the course of the trial.  Each participant will be followed up for a minimum of 15 months, but up to 24 months.  Treatment will last for three months from the date of randomisation. |
| Primary End –points: | Successful eradication of *P. aeruginosa* infection three months after treatment has started, remaining infection free through to 15 months after the start of allocated treatment. |
| Secondary End-points: | 1. Time to reoccurrence of original *P. aeruginosa* infection. 2. Re-infection with a different genotype of *P. aeruginosa* 3. Lung function 4. Oxygen saturation 5. Growth and nutritional status – height, weight and body mass index 6. Number of pulmonary exacerbations 7. Admission to hospital 8. Number of days spent as an inpatient in hospital over the three-month period post-randomisation and between three months and 15 months post-randomisation (other than 14 days spent on initial Intravenous treatment) 9. Quality of life 10. Utility 11. Adverse events 12. Other sputum/cough microbiology (Methicillin resistant *Staphylococcus aureus*, *Burkholderia cepacia* complex, Aspergillus, Candida Infection) 13. Cost per patient (from NHS perspective) 14. Incremental cost effectiveness ratio (cost per successfully treated patient, cost per Quality Adjusted Life Year) 15. Carer burden (absenteeism from education or work) 16. Participant burden (absenteeism from education or work) |
| Statistical Methods: | The primary analysis will use the principle of intention to treat based on all randomised participants, as far as practically possible.  For a participant to have a ‘success’ on the primary outcome, they will need to have had their initial *P. aeruginosa* infection eradicated following treatment at three months and remain infection free until 15 months following the start of allocated treatment. *P. aeruginosa* eradication will be reported in terms of relative risk of success and its 95% confidence interval.  For the secondary outcomes, continuous data will be reported as a difference in means and binary data will be reported in terms of the relative risk each with 95% confidence intervals.  Time to reoccurrence of *P. aeruginosa* will be summarised by Kaplan-Meier curves for each treatment group and compared overall using log rank tests and survival regression methods.  Missing data will be monitored and strategies developed to minimise its occurrence.  Missing data will be handled by considering the robustness of the complete case analysis to sensitivity analyses using various imputation assumptions.  However, these will be informed by data collected on the reasons for missing data. |
